# Type 3 immunity: a perspective for the defense of the mammary gland against infections

**DOI:** 10.1186/s13567-020-00852-3

**Published:** 2020-10-15

**Authors:** Pascal Rainard, Patricia Cunha, Rodrigo P. Martins, Florence B. Gilbert, Pierre Germon, Gilles Foucras

**Affiliations:** 1 ISP, INRAE, Université de Tours, UMR1282, Tours, Nouzilly France; 2 IHAP, Université de Toulouse, INRAE, ENVT, Toulouse, France

**Keywords:** type 3 immunity, IL-17, Th17, mastitis, dairy ruminants, mammary gland

## Abstract

Type 3 immunity encompasses innate and adaptive immune responses mediated by cells that produce the signature cytokines IL-17A and IL-17F. This class of effector immunity is particularly adept at controlling infections by pyogenic extracellular bacteria at epithelial barriers. Since mastitis results from infections by bacteria such as streptococci, staphylococci and coliform bacteria that cause neutrophilic inflammation, type 3 immunity can be expected to be mobilized at the mammary gland. In effect, the main defenses of this organ are provided by epithelial cells and neutrophils, which are the main terminal effectors of type 3 immunity. In addition to theoretical grounds, there is observational and experimental evidence that supports a role for type 3 immunity in the mammary gland, such as the production of IL-17A, IL-17F, and IL-22 in milk and mammary tissue during infection, although their respective sources remain to be fully identified. Moreover, mouse mastitis models have shown a positive effect of IL-17A on the course of mastitis. A lot remains to be uncovered before we can safely harness type 3 immunity to reinforce mammary gland defenses through innate immune training or vaccination. However, this is a promising way to find new means of improving mammary gland defenses against infection.

## A novel approach is required to improve mastitis vaccines

Mastitis is the most frequent and economically important disease of dairy ruminants worldwide [1]. The main bacteria responsible for mammary gland (MG) infections (staphylococci, streptococci, and coliform bacteria) can cause acute clinical mastitis, but more often subclinical long-lasting infection and inflammation [2]. Both types of infections are characterized by the recruitment of leukocytes, mainly neutrophils, into the mammary tissue and secretions. The mechanisms behind this recruitment are incompletely defined, and although several MG defenses have been identified, their coordination and regulation at the cellular and molecular levels are insufficiently understood. Besides, despite many attempts at devising efficacious vaccines, those presently licensed do not fulfill all expectations [2, 3]. We need novel approaches that could offer new development perspectives. We propose that putting the stress on type 3 immunity, its signature cytokines and the cells that produce them or respond to them, could help fulfill this need. In this position paper, we present the reasons that make type 3 immunity a likely major mechanism of MG defense against infection, and we describe the observational and experimental data that support this view. We highlight the emerging knowledge that suggests a role for type 3 immunity both in the inflammatory response of the MG to infection and the prospects that this outlook creates for our understanding of mastitis pathogenesis and its control through vaccination. Vaccination favoring type 3 immunity is an active field of investigation in medical research [4, 5]. We plead for its consideration in the mastitis vaccine field. The recent development of new tools allows researchers to investigate this new research area for dairy ruminants, and to fill some of the knowledge gaps that hamper mastitis control.

## Type 3 immunity: a defense mechanism at epithelial barriers

The immune system comprises different classes of effector immunity. The term type 3 immunity emerged recently [6] to qualify an immune response that complements the type 1 and type 2 immunity, heralded by the Th1 and Th2 lymphocytes, respectively. As type 3 immunity is associated with Th17 differentiation and effector functions, it has initially been called type 17 immunity, but type 3 immunity seems more convenient, also encompassing the innate arm of this kind of immunity, mediated by type 3 innate lymphoid cells (ILC3).

Type 3 immunity can be characterized by an immune response that exhibits a distinct profile that includes expression of the genes encoding interleukin-17A (IL-17A), IL-17F and IL-22, and key transcription factors retinoic acid-related orphan receptor Rorγt and Rorα and their gene targets [6, 7]. Type 3 immunity is an ancient immune mechanism whose roots can be found in nematodes or mollusks, which evolved to bridge innate and adaptive immunity to help metazoans live in a microbe’s world [8]. Type 3 immunity is characterized by the recruitment of neutrophils and the stimulation of epithelial antimicrobial defenses at infection sites. Type 3 immunity is often triggered by extracellular bacteria and fungi and seems to be particularly suited to defend epithelial barriers against these pathogens. It can also be implicated in chronic inflammation and autoimmunity. The cells responsible for type 3 immunity are diverse, including ILC3, γδT cells, CD4 helper (Th17) and CD8 (Tc17) αβT cells. Type 3 immune responses deal with infections through the collaboration between antigen-presenting cells, pathogen-specific B and T cells, innate lymphoid cells, neutrophils, and epithelial cells, thus orchestrating the interplay of innate and adaptive immune components [9].

Surprisingly, the MG is not considered as an organ protected by type 3 immunity, although there are considerations about the MG that make it a very likely type 3 battleground for invading bacteria (Table 1). This omission likely stems from the shortfall in the immunological toolkit for the study of type 3 immunity in ruminants and the relative disinterest for infectious mastitis in medical research. However, the toolbox for ruminant immunology was recently enriched, which should enable advances in this research field [10, 11]. Recently, CD4 + cells producing IL-17A have been described in ruminants [10, 12], and bovine Th17 cells have been isolated and expanded in culture [11].

**Table 1:** Theoretical reasons why type 3 immunity should contribute to MG defense against pathogens.

Features of type 3 immunity	Features of mammary gland defenses	Ref
Immunity to extracellular bacteria and fungi	Infection by extracellular bacteria	[61]
Defense of epithelial barriers	Mainly epithelial infection (“duct disease”)	[62]
Amplifies neutrophilic inflammation	Neutrophils main cell type recruited during mastitis	[63]
Neutrophils important effector arm of type 3 immunity	Neutrophils main immune defense of the mammary gland	[63]
Induces epithelial self-defense by antimicrobial peptides	Mammary epithelial cells produce AMPs in response to bacteria or cytokines	[62]
Targets epithelial cells to trigger inflammation (chemokines)	Mammary epithelial cells respond to IL-17A by secreting chemokines	[17, 24]
Signature cytokines: IL-17A, IL-17F, IL-22	IL-17A, IL-17F, IL-22 in mastitic milk	[19, 23]
Targets epithelial cells through receptors to IL-17 and IL-22	Mammary epithelial cells express IL-17R and respond to IL-17A & IL-17F	[24]
Immunization elicits CD4 + cells producing IL-17 (Th17 lymphocytes)	CD4 + IL-17A + cells correlate with vaccination or antigen-specific sensitization of the mammary gland	[33]
The IL-23/IL-17 axis drives granulopoiesis	Mastitis drains neutrophil reserves	[64]

## What we know about type 3 immunity in the mammary gland

The production of IL-17 in the MG has been reported in several studies. Following the characterization of the bovine IL-17A cDNA [13], increases in *Il17* gene transcripts measured by RT-qPCR in tissue and milk leukocytes from bovine MG infected by *Staphylococcus aureus* or *Streptococcus uberis* provided observational and circumstantial evidence to suggest that IL-17 was implicated in the defense of the MG [13–16]. Overexpression of genes encoding IL-17A and IL-17F was found in the mammary tissue during infection by *E. coli* [17]. When ELISAs for bovine IL-17 and IL-22 became available, IL-17A, IL-17F, and IL-22 were found in the milk of cows infected by *E. coli* [18, 19] or goats infected by *S. aureus* [20].

It has been established that IL-17A contributes to the defense of the MG against infections. Experimental evidence came from mouse models of MG infection. Experimental infection with a mastitis *S. aureus* strain of goat origin revealed an early influx of γδT cells producing IL-17A into the MG [21]. Other experimental infections of mouse MG with either *S. aureus* or *E. coli* showed an early contribution of IL-17 and Th17 cells to the control of infection, rapidly followed by IL-10 and probably regulatory T cells (Treg) intervention [22, 23]. In those studies, co-administration of IL-17A along with the inoculum increased the recruitment of neutrophils and decreased the severity of infection, whereas the administration of an antibody blocking IL-17A decreased the recruitment of neutrophils and resulted in an increased *E. coli* bacterial load. Those studies demonstrated that IL-17A plays a part in the defense of the mouse MG, with clear beneficial effects in the case of *E. coli* mastitis, and moderate effects on *S. aureus* mastitis. Of note, IL-22 concentrations increased markedly in infected MGs and the depletion of γδT lymphocytes did not affect the *E. coli* mammary load [23].

Another finding was that IL-17A amplifies mammary epithelial cells (MECs) responses to infection. A major effector arm of type 3 immunity involves epithelium proinflammatory and antimicrobial responses. Bovine MECs express (mRNA) the two components of the IL-17 receptor, IL17RA, and IL-17RC, and they respond to IL-17A or IL-17F by producing chemokines and antimicrobial peptides [24]. Interestingly, the response of MECs was enhanced by the simultaneous exposure to TNF-α or to staphylococcal or *E. coli* microbe-associated molecular patterns (MAMPs), suggesting that IL-17 exerts its full potential in a context of inflammation triggered by bacteria [17, 24]. Of note, under these conditions, MECs markedly overexpressed (mRNA) CCL20, a chemokine that attracts cells expressing the receptor CCR6, which include most of type 3 immunity cells [25]. This chemokine was found at the protein level in milk from bovine MGs exposed to *E. coli* LPS [26].

The preceding data refer to the contribution of type 3 immunity through its innate arm, even though Th17 cells were involved in the mouse mastitis model [23]. These Th17 cells may be the MG counterpart of the innate Th17 cells that have been found to play a part in the immune response to intestinal bacterial pathogens in mice [27]. The contribution to adaptive immunity is less well established. However, there is some evidence that type 3 immunity can be induced in the MG by vaccination. Neutrophilic inflammation in response to the local infusion of antigens can be induced in the MG by immunization. This mammary antigen-specific reaction (mASR) was first described by using ovalbumin as a model antigen. Upon infusion of a few µg of ovalbumin through the teat canal of cows previously sensitized to this antigen by subcutaneous immunization, neutrophils flocked to the lumen of the MG, whereas control unimmunized cows did not react [28]. The same phenomenon was reproduced in the MG of guinea pigs with killed *S. aureus* as antigen [29]. Experiments with adoptively sensitized guinea pigs have shown that lymphocytes, but not immune serum, made the recipient animals responsive to the sensitizing antigen [30, 31]. In the milk of sensitized and antigen challenged cows, IL-17A and IFN-γ were found as soon as 8 h post-challenge, along with overexpressed transcripts of the genes encoding IL-17A, IL-17F, IL-21, IL-22, IL-26, and IFN-γ in mammary tissue [32]. The mASR was later shown to correlate with the induction of circulating CD4 T cells producing both IL-17A and IFN-γ [33]. Indeed, a whole-blood assay measuring the production of IL-17A and IFN-γ upon stimulation with the antigen correlated with the magnitude of mASR [32]. Overall, these results strongly suggest that Th17 lymphocytes are associated with antigen-specific neutrophilic inflammation in the MG. This immune response supposes the existence of antigen-presenting cells (APC) in the MG. Cells with a CD11c high, MHCII + and CD205 + phenotype have been described in the bovine MG, within the alveolar epithelium and the connective tissue [34]. These cells, resembling dendritic cells, are distinct from macrophages and in a position to sample the lumen of the MG and present antigenic peptides to tissue-resident effector lymphocytes.

Mirroring the synergy of bacterial MAMPs with IL-17A seen in vitro with MECs, a synergy between innate (MAMPs) and adaptive (Th17) immunity seems to operate in vivo to amplify neutrophilic inflammation in the MG [35]. Such a synergy that increases the recruitment of neutrophils at the onset of infection may reduce the bacterial burden, facilitate the prompt clearance of bacteria, and consequently reduce the initial inflammatory insult to mammary tissue. This scenario was elicited by immunizing cows with a surface protein of *Streptococcus agalactiae* [36]. This result deserves further investigation at a larger scale and in greater depth with the immunological toolbox currently available for ruminants. An attempt was recently made by vaccinating cows with *E. coli* extracts before intramammary challenge with the vaccinating strain [19]. The results indicated a level of protection involving the production of IFN-γ and possibly Th17 cells, but the improvement over control cows was moderate. The antibody response did not seem to play an important role in the improved response to MG infection. A further study indicated that a type 3 immunity had been induced in the mammary tissue by local vaccination, as evidenced by the transcriptomic profile of CD4 T cells isolated from the MG parenchyma 24 h post-infection [37].

**Figure 1.**
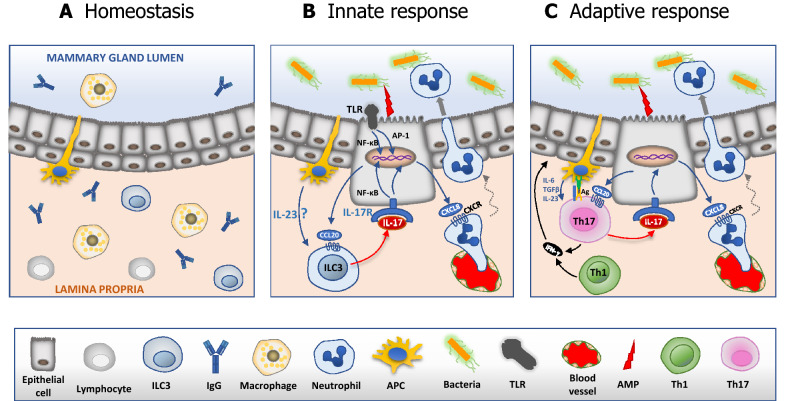
**Schematic view of type 3 immunity governing neutrophilic inflammation in the infected mammary gland.** **A** The healthy MG is an immunologically quiet place at homeostasis as there is little or no bacterial stimulation. **B** According to the innate immunity scenario, macrophages (MΦ) or epithelial cells responding to invading bacteria attract and stimulate ILC3 that respond by secreting IL-17A. In turn, epithelial cells recruit neutrophils through chemokine secretion. **C** In the adaptive immunity scenario, the capture of bacteria and presentation of antigen by an antigen-presenting cell (APC) to a tissue resident Th17 cell triggers the release of IL-17A that prompts epithelial cells to secrete chemokines (including CXCL8). These chemokines recruit neutrophils that cross the epithelium to reach invading bacteria.

Undoubtedly, there is still a lot to be done before adaptive type 3 immunity can be harnessed effectively to protect against mastitis. However, a putative schematic view of the ways type 3 immunity might operate in the MG can be envisioned (Figure 1).

## What we do not know: knowledge gaps

A major issue is the identification of the cells that could contribute to the defense of the MG against infections. Several different cell types are enrolled under the banner of type 3 immunity and produce the signature cytokines. There are antigen-specific cells with αβT-cell receptor (TCR) CD4 + (Th17) and CD8 + T (Tc17) or γδTCR (γδT cells), and lymphoid cells that do not express a TCR, the ILCs. Lymphocytes of the CD4 and CD8 lineages with memory phenotype are found in the milk of healthy and infected MGs, but their functions remain speculative [38, 39]. Among these cells, CD4 T cells expressing RORγt or producing IL-17A have been found in healthy or infected, lactating or involuting mouse MGs, but RORγt + CD8 + T cells have not yet been reported [23, 40]. These lymphocytes require antigen presentation by APCs in association with MHC class I or class II to be fully activated. Th17 cells are well known for their plasticity, and their changing phenotype depends on their environment. The cells, metabolites and cytokines that could influence the phenotype of Th17 cells in the MG remains an unexplored but important area of research. Besides Th17 cells, γδT cells express receptors of the innate immune system such as Toll-like receptors (TLR)1, TLR2, TLR3, TLR4 and dectin 1, which allow them to respond to MAMPs [41, 42]. They can also secrete IL-17A and IL-22 without the engagement of the TCR in the presence of IL-1β and IL-23. In the bovine species, WC1 + γδT cells, CD4 + (Th17) and CD8 + T cells have been shown to produce IL-17A [10, 12, 43, 44]. In organs and at periphery tissues, most bovine γδT cells are of the WC1-phenotype and would differ functionally from the WC1 + phenotype [41]. During infection, γδT cells are recruited in milk and a few studies indicated selective recruitment of particular subsets [45, 46]. Other type 3 immune cells do not possess TCR and belong to the innate arm of immunity. The most recently described are the ILCs that come in three types, ILC1, ILC2 and ILC3 [47]. They are the innate counterparts of the Th1, Th2, and Th17 adaptive T cells. ILC3 cells mostly populate parenchymal tissues and mucosal epithelia, where they hold important functions of early resistance to pathogens, regulation of inflammation and tissue homeostasis [48]. Bovine ILCs have not been described yet. These cells are difficult to study because they reside mainly in peripheral tissues, hardly recirculate and are difficult to extract from their niche environment. We do not know if they can respond to MAMPs, as human ILCs do, or cannot, like mice ILCs. ILC3 respond to IL-23 and IL-1α or IL-1β to produce their effector cytokines (IL-17A, IL-17F, IL-22) [48]. Whether IL-23 is overexpressed in the MG during infection remains to be established. It is likely that ILCs are present in mammary tissue and contribute to the defense of the MG, but so far that has not been documented.

We do not know precisely which cells produce IL-17A/F and IL-22 in the MG during infection. Much of the IL-17 released during an inflammatory response is produced by innate immune cells [49]. In both mice and humans, γδT cells and ILCs are important sources of the Th17 cytokines IL-17A, IL-17F and IL-22 in the epithelial tissues. We can speculate that different cell types secrete IL-17A and IL-22 during an *E. coli* infection or LPS inflammation episode because IL-17A/F and IL-22 concentration increases in milk did not coincide [19]. We are also ignorant of the respective roles of the type 3 immunity signature cytokines in the defense of the MG against infection, wound healing, physiology at involution and homeostasis. We know that IL-22 is endowed with important functions of control of pathogens and tissue repair [50]. However, we do not know the effects of IL-22 on mammary tissue. Several studies have shown that in the bovine species type 3 immunity and the associated cytokines are likely to play a positive or negative part in viral, mycobacterial (tuberculosis and paratuberculosis) or parasitic diseases [43, 51–55]. The cytokine IL-26 is also associated with type 3 immunity [56]. Contrary to the laboratory mouse, but like humans, ruminants have a functional *Il26* gene. Human IL-26 possesses antibacterial activity [57]. Bovine *Il26* can be expressed in mammary tissue and by bovine Th17 cells [11, 32], but its role in the mastitis context remains to be established.

Another knowledge gap refers to the homing and addressing of innate and adaptive lymphocytes to mammary tissue. Few studies addressed this important issue in the MG of ruminants. What we know is that the adhesion molecule MAdCAM-1 was not found to be expressed in the bovine MG and supramammary lymph nodes whatever the physiological stage and lymphocytes expressing the counter-receptor α4β7 were not detected in mammary tissues [58], suggesting that this vascular addressin is not involved in the recruitment of lymphocytes in healthy glands.

## Prospects: beyond the mastitis vaccine deadlock

We have seen that there are several theoretical, observational and experimental arguments supporting the notion that type 3 immunity is an important arm of the immune defense of the MG. It is patent that a lot remains to be uncovered about the ins and outs of this immune type in ruminant in general and in the MG in particular. In the mastitis context, a major driver for a better knowledge of type 3 immunity in the MG is the development of efficacious vaccines. Conventional views of MG defenses and vaccine mode of action, which are essentially based on antibody response, may have come to a deadlock. An alternative approach based on new developments of immunology is possible by capitalizing on a better knowledge of cell-mediated type 3 immunity. This raises the possibility of new experiments and progress towards more efficacious vaccines, by combining immunology knowledge and vaccinology approaches [59].

For practical purposes, what do we need to harness type 3 immunity to control mastitis? The issue of the orientation of the immune response in ruminants is crucial. A major research topic should be the search for antigens and adjuvants that engage the appropriate APC and ILC subsets and therefore orient the adaptive immune response towards type 3 immunity. Preliminary data indicate that it is possible to induce protective Th17 cells in the MG. To elicit a protective type 3 immune response in the MG, we will need to select antigens with T-cell epitopes presented by the MHC class II targeting the right APCs by the proper delivery system, with the appropriate adjuvant. Undoubtedly, much remains to be explored before we are able to meet these different requirements. We know very little about CD8 T cells producing IL-17 (Tc17). Yet, those cells might be instrumental in flushing out bacteria sheltered in epithelial cells or macrophages. Other cells of the innate arm of type 3 immunity are also likely to play an important part in the defense of the MG. It can also be envisaged harnessing the innate component of type 3 immunity with immunomodulators. A foreseeable complication to these approaches will come from the proinflammatory facet of type 3 immunity and its impact on the MG integrity. In effect, an overshooting inflammatory reaction could jeopardize the MG function, i.e., secretion of milk. The immune response must be adapted to the pathogen: what is good for *E. coli* mastitis may not be necessarily good for *S. uberis* or *S. aureus* mastitis. Type 3 immunity could be beneficial by improving the efficiency of the acute phase of an infection that self-cures as *E. coli* mastitis usually does. Its role may be more complex in chronic infections such as *S. aureus* mastitis. It may even be detrimental in the case of *S. uberis* infection, as the efficiency of phagocytic killing by neutrophils is dubious [60]. However, IL-17 correlated with the resolution of MG infections by *S. uberis* [14], an observation that could be in relation with type 3 immunity protective mechanisms other than neutrophilic inflammation, such as the contribution of Tc17 cells or the self-defense response of epithelial cells. Type 1 immunity or Tc17 cells may be a more important component of the immune response with bacteria that are apt at surviving within epithelial cells such as *S. aureus* than with *E. coli*. Taking into account the pathogenesis of the infection at issue will be necessary. Moreover, inflammation-driven dysfunction may be more or less critical according to the organ at stake. In the lungs, for example, loss of function is incompatible with life, whereas, in the mammary gland, a temporary loss of function is critical only to the offspring if it lasts several days, and thus a level of inflammation is tolerable in the MG that would not be in the lungs. However, this caveat should be addressed by a fine-tuning of the elicited immunity. The new tools and concepts of immunology will help to respond to these challenges.

## Data Availability

Not applicable.

## Abbreviations

APC: antigen-presenting cell
IL: interleukin
ILC: innate lymphoid cell
LPS: lipopolysaccharide
mASR: mammary antigen-specific reaction
MAMP: microbe-associated molecular pattern
MEC: mammary epithelial cell
ROR: RAR-related orphan nuclear receptor
TLR: toll-like receptor
